# Loss of PUMA protects the ovarian reserve during DNA-damaging chemotherapy and preserves fertility

**DOI:** 10.1038/s41419-018-0633-7

**Published:** 2018-05-23

**Authors:** Quynh-Nhu Nguyen, Nadeen Zerafa, Seng H. Liew, F. Hamish Morgan, Andreas Strasser, Clare L. Scott, Jock K. Findlay, Martha Hickey, Karla J. Hutt

**Affiliations:** 1 Department of Obstetrics and Gynaecology, The University of Melbourne, Parkville, VIC Australia; 2 Development and Stem Cells Program, Monash Biomedicine Discovery Institute, and Department of Anatomy and Developmental Biology, Monash University, Clayton, VIC Australia; 3 The Walter and Eliza Hall Institute of Medical Research, Parkville, VIC Australia; 4 Department of Medical Biology, University of Melbourne, Parkville, VIC Australia; 5 Centre for Reproductive Health, Hudson Institute of Medical Research, Clayton, VIC Australia; 6 Monash University, Clayton, VIC Australia; 7 The Royal Womens Hospital, Parkville, VIC 3052 Australia

## Abstract

Female gametes are stored in the ovary in structures called primordial follicles, the supply of which is non-renewable. It is well established that DNA-damaging cancer treatments can deplete the ovarian reserve of primordial follicles, causing premature ovarian failure and infertility. The precise mechanisms underlying this chemotherapy-driven follicle loss are unclear, and this has limited the development of targeted ovarian-protective agents. To address this fundamental knowledge gap, we used gene deletion mouse models to examine the role of the DNA damage-induced pro-apoptotic protein, PUMA, and its transcriptional activator TAp63, in primordial follicle depletion caused by treatment with cyclophosphamide or cisplatin. Cyclophosphamide caused almost complete destruction of the primordial follicle pool in adult wild-type (WT) mice, and a significant destructive effect was also observed for cisplatin. In striking contrast, *Puma*^−/−^ mice retained 100% of their primordial follicles following either genotoxic treatment. Furthermore, elimination of PUMA alone completely preserved fertility in cyclophosphamide-treated mice, indicating that oocytes rescued from DNA damage-induced death can repair themselves sufficiently to support reproductive function and offspring health. Primordial follicles were also protected in *TAp63*^−/−^ mice following cisplatin treatment, but not cyclophosphamide, suggesting mechanistic differences in the induction of apoptosis and depletion of the ovarian reserve in response to these different chemotherapies. These studies identify PUMA as a crucial effector of apoptosis responsible for depletion of primordial follicles following exposure to cyclophosphamide or cisplatin, and this indicates that inhibition of PUMA may be an effective ovarian-protective strategy during cancer treatment in women.

## Introduction

Female gametes are stored in the ovary in structures called primordial follicles, each of which contains an immature oocyte encased in a single layer of somatic cells known as granulosa cells. All mature hormone-producing follicles and oocytes for ovulation, and hence conception, are derived from the pool of primordial follicles present in the ovaries at birth, making their number a critical determinant of future fertility and ovarian endocrine function^1,2^. Throughout reproductive life, the number of primordial follicles slowly declines as they commence growth, resume meiosis and are ovulated, or more commonly because they undergo atresia and die. Age-related infertility, and subsequently menopause, occur once the supply of primordial follicles has been exhausted^3^. Crucially, it is not possible to make new primordial follicles after birth, even if the supply is prematurely depleted^4–7^.

Within primordial follicles, oocytes exist in a unique biologic stasis: they are non-growing, non-dividing and have initiated meiosis, but remain in diplotene arrest until recruited into the growing follicle pool, often much later in life. This extreme longevity and arrested state may make primordial follicle oocytes particularly vulnerable to genotoxic stress^8^, and thus it is crucial that they are subject to rigorous surveillance, with prompt detection and repair of DNA damage, or elimination of oocytes by apoptosis when genomic integrity is critically compromised. DNA-damaging cancer therapy, including chemotherapy and radiotherapy, is a prime example of primordial follicle depletion due to genotoxic insult, and is the single most common cause of acquired primary ovarian insufficiency in girls and young women^8–10^. Despite an increase in the understanding of the risk that chemotherapeutic drugs pose to the ovary and female fertility, currently no effective, non-invasive, adjuvant therapies exist to prevent ovarian damage during cancer treatment.

Of the major classes of chemotherapeutic drugs, the alkylators are known to be the most damaging to ovaries and fertility, followed by the anthracyclines and platinum-based alkylating-like agents^11^. Alkylators, such as cyclophosphamide, and platinum compounds, such as cisplatin, exert their anticancer effects via differing mechanisms to cause inter-strand DNA crosslinks. This results in the formation of double-stranded DNA breaks, leading to cell death if these defects are not repaired^12^. Indeed, both cyclophosphamide and cisplatin have been shown to damage the oocytes of primordial follicles in mice and human ovarian tissue^13–17^ and directly deplete the ovarian primordial follicle pool in a dose-dependent manner^18–20^, although the apoptotic regulators of this process have not been fully characterized.

PUMA is a member of the pro-apoptotic BH3-only sub-group of the BCL-2 protein family^21,22^. PUMA is critical for the initiation of p53-mediated apoptosis by activating the pro-apoptotic BCL-2 family members, BAX and BAK, either by direct binding, or indirectly via inhibition of pro-survival BCL-2 family members^23–25^. We have previously shown that PUMA exerts similar actions in the ovary, where it is an essential effector of TAp63-mediated apoptosis of primordial follicle oocytes in response to DNA damage caused by γ-irradiation. Remarkably, female mice deficient in PUMA, or PUMA and NOXA (another pro-apoptotic BH3-only protein), are substantially protected from irradiation-induced primordial follicle apoptosis and remain fertile^26^. However, the role of PUMA in the DNA damage response elicited in oocytes by chemotherapeutic drugs commonly used to treat cancers in women of reproductive age have not been studied.

The current study aimed to answer this uncertainty by utilizing genetic mouse models of PUMA or TAp63 loss to definitively characterize the role of these two proteins in ovarian reserve depletion induced by cyclophosphamide and cisplatin. We also sought to determine whether blocking apoptosis mediated by this pathway represents a potential target for the development of new fertility preservation strategies in female cancer patients treated with alkylating agents.

## Results

### Treatment with cyclophosphamide or cisplatin significantly depletes the ovarian reserve

**Fig. 1:**
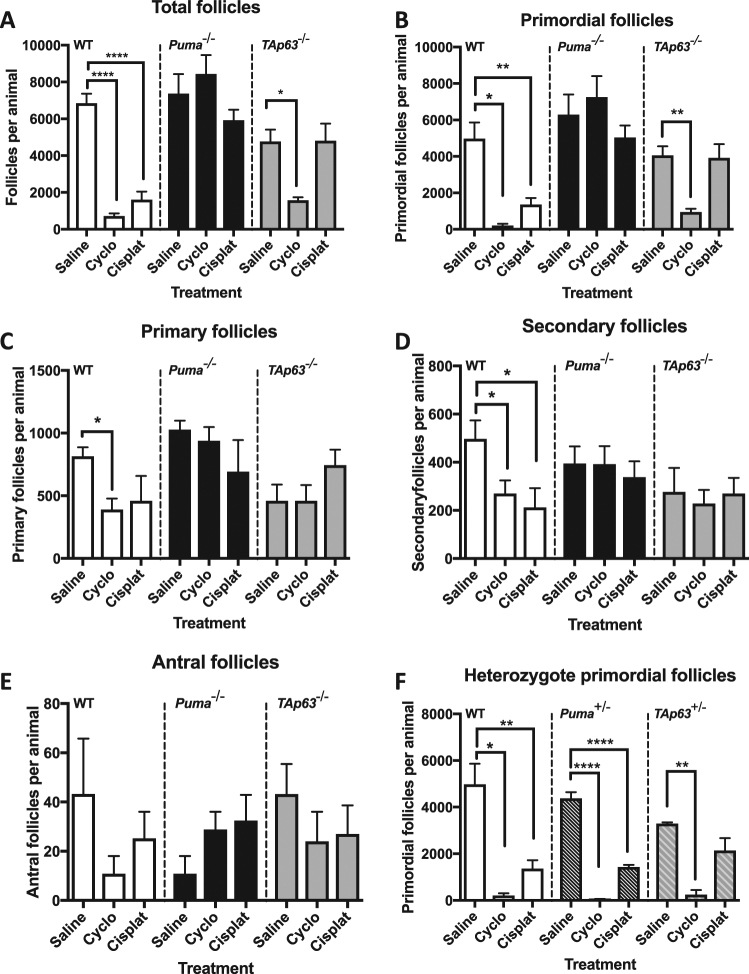
Follicular quantification by genotype and treatment group. Total (**a**), primordial (**b**), primary (**c**), secondary (**d**), and antral follicles (**e**) were counted in WT, *Puma*^−/−^ and *TAp63*^−/−^ female mice treated with saline, cyclophosphamide or cisplatin. Primordial follicles were counted in *Puma*^*+/-*^ and *TAp63*^*+/-*^ heterozygote mice and compared with WT controls (**f**). Data are expressed as mean follicles per animal + SEM; *p ≤ 0.05, **p ≤ 0.01, ****p ≤ 0.0001 compared with genotype-matched saline control mice (one-way ANOVA, Tukey’s multiple comparisons test). For clarity, only selected comparisons are shown

To assess the impact of cyclophosphamide and cisplatin on the ovarian reserve in vivo, we treated adult female mice with a single dose of saline (negative control), cisplatin (5 mg/kg) or cyclophosphamide (300 mg/kg) by intraperitoneal injection; dosing was determined based on regimens previously used successfully to treat cancers in mice^27–31^. Ovaries were then harvested for follicle quantification 5 days later. In keeping with what is already known about the relative toxicities of these treatments^32^, an analysis of total follicle numbers showed that cyclophosphamide and cisplatin both caused a massive net loss of follicles in WT females (WT saline: 6852 ± 510 vs WT cyclophosphamide: 720 ± 141, *p* < 0.0001; vs WT cisplatin: 1616 ± 429, *p* < 0.0005) (Fig. 1a).

To understand which stages of follicular development were impacted by chemotherapy, we determined the numbers of follicles within the primordial and growing (primary, secondary and antral) follicle populations. We found that treatment with cyclophosphamide dramatically reduced the number of primordial follicles in WT females, with only 4% of primordial follicles surviving (Fig. 1b). Exposure to cyclophosphamide also resulted in significant depletion of primary (48% survival; *p* < 0.05 versus saline-treated WT) (Fig. 1c) and secondary follicles (54% survival; *p* < 0.05 versus saline-treated WT) (Fig. 1d). Similar to treatment with cyclophosphamide, significant depletion of the primordial follicle reserve was observed in WT females exposed to cisplatin, with only 27% of primordial follicles remaining (Fig. 1a). Similarly, exposure to cisplatin caused significant secondary follicle loss (48% survival, *p* < 0.05 versus saline-treated WT) (Fig. 1d). There was also a trend toward primary follicle depletion, although this did not reach statistical significance (Fig. 1c). Neither antral follicle nor corpora lutea numbers were impacted by chemotherapy treatment (Fig. 1e; Fig. S2, Additional File 1).

### Loss of PUMA protects the ovarian reserve from cyclophosphamide or cisplatin

We then went on to investigate whether loss of PUMA would confer protection on the primordial follicle pool from cyclophosphamide- or cisplatin-induced depletion. Remarkably, *Puma*^−/−^ mice treated with cyclophosphamide retained 100% of their primordial follicles (*Puma*^−/−^ saline: 6294 ± 1103 vs *Puma*^−/−^ cyclophosphamide: 7252 ± 1150, *p* = 0.58) (Fig. 1b). This reveals that PUMA is required for primordial follicle loss after DNA damage caused by cyclophosphamide. A similar protective effect was also seen in *Puma*^−/−^ females treated with cisplatin (*Puma*^−/−^ saline: 6294 ± 1103 vs *Puma*^−/−^ cisplatin: 5035 ± 662.5, *p* = 0.38) (Fig. 1b). Loss of PUMA also completely prevented the depletion of primary and secondary follicles caused by cyclophosphamide treatment and the depletion of secondary follicles caused by cisplatin treatment (Figs. 1c, d). In keeping with these data for individual follicle stages, total follicle numbers were similar in saline, cyclophosphamide and cisplatin-treated *Puma*^−/−^ females, demonstrating complete protection of the total follicular complement (Fig. 1a).

### Loss of TAp63 protects the ovarian reserve from cisplatin treatment but not cyclophosphamide

Previous studies have indicated that TAp63 is activated in oocytes following DNA damage induced by γ-irradiation or cisplatin^14,33^, and that following irradiation, it acts by transcriptionally activating the BH3-only genes *Puma* and *Noxa*^26^. However, the role of TAp63 in oocytes following DNA damage induced by cyclophosphamide has not been investigated. Consistent with these earlier reports, we found that *TAp63*^−/−^ mice were completely protected following cisplatin treatment (100% survival; *TAp63*^−/−^ saline: 4060 ± 497 vs *TAp63*^−/−^ cisplatin: 3918 ± 754, *p* = 0.98) (Fig. 1b). Strikingly, however, only 26% of primordial follicles in *TAp63*^−/−^ mice survived after treatment with cyclophosphamide (*TAp63*^−/−^ cyclophosphamide: 957 ± 171, *p* < 0.05) (Fig. 1b). The marked difference in the number of surviving primordial follicles in *TAp63*^−/−^ females as compared with the *Puma*^−/−^ females after treatment with cyclophosphamide indicates that PUMA is indeed crucial for cyclophosphamide-induced primordial follicle death, but that cyclophosphamide-induced follicle loss can occur independently of TAp63. Primary, secondary and antral follicle numbers (Figs. 1b-d) were similar in saline-, cisplatin- and cyclophosphamide-treated *TAp63*^−/−^ females.

Total follicle counts in *TAp63*^−/−^ females followed the pattern seen in primordial follicles, with a large net loss of follicles observed in cyclophosphamide-treated *TAp63*^−/−^ mice (*TAp63*^−/−^ saline: 4767 ± 649 vs *TAp63*^−/−^ cyclophosphamide: 1577 ± 161; *p* < 0.05) (Fig. 1a). No significant difference was seen between total follicle counts in saline-treated *TAp63*^−/−^ females vs their cisplatin-treated counterparts (*TAp63*^−/−^ cisplatin: 4813 ± 923, *p* = 0.999) (Fig. 1a).

### Protection of the ovarian reserve requires complete loss of PUMA

To assess whether partial loss of PUMA or TAp63 would prevent chemotherapy-induced depletion of the primordial follicle pool, the study was repeated in *Puma*^*+/−*^ and *TAp63*^*+/−*^ heterozygote mice. *Puma*^+/−^ females were not protected from primordial follicle depletion after treatment with either cisplatin or cyclophosphamide when compared with saline-treated controls (*Puma*^+/−^ saline 4385 ± 259 vs *Puma*^+/−^ cyclophosphamide 40 ± 23, *p* < 0.0001; vs *Puma*^+/−^ cisplatin 1436 ± 86, *p* < 0.0001) (Fig. 1f). This suggests that the protection conferred by PUMA loss is dependent upon complete knockout, or at least >50% reduction in PUMA. In contrast, primordial follicle counts of *TAp63*^+/−^ mice showed that partial loss of TAp63 may confer protection for the primordial follicle pool against cisplatin (Fig. 1f).

### Rescued primordial follicles are morphologically normal

**Fig. 2:**
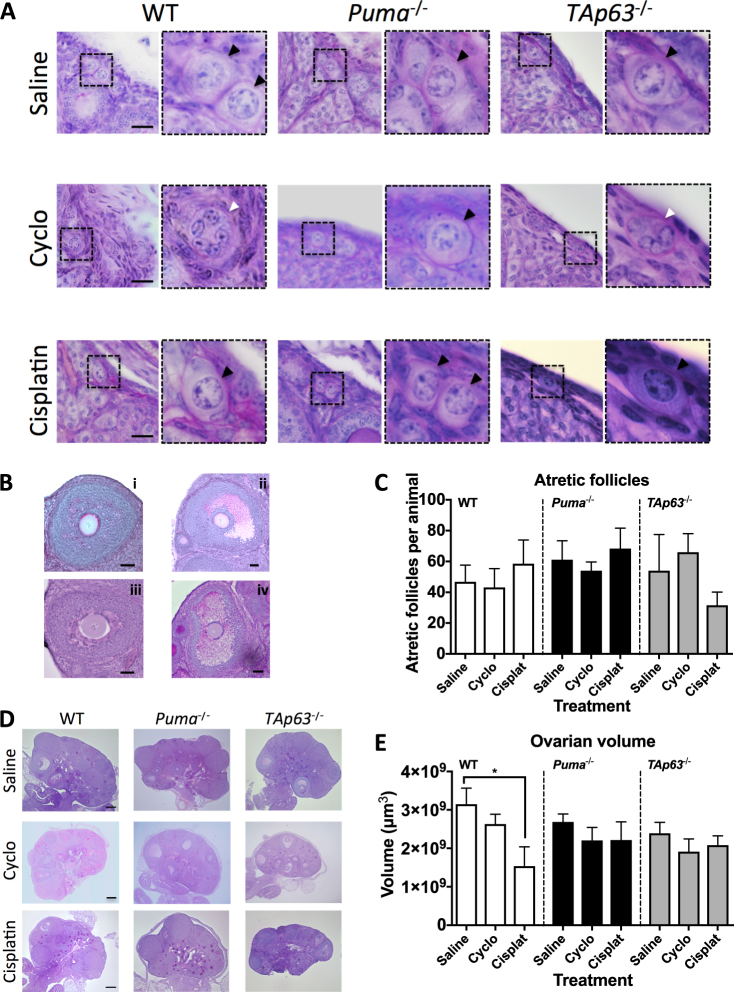
Follicular morphology. **a** Representative images of PAS-stained ovarian cortex from mice of the indicated genotypes harvested 5 days following treatment with saline, cyclophosphamide or cisplatin. Dashed inset boxes denote areas shown in higher magnification to the right. Black arrowheads denote primordial follicles. White arrowheads denote residual follicular structures without a surviving central oocyte. Scale bar = 20 µm. **b** Representative images of healthy pre-antral (i), healthy antral (ii), atretic pre-antral (iii) and atretic antral follicles (iv). Scale bars = 50 µm. **c** Quantification of atretic follicles (per animal; *n* = 4–5). Data expressed as mean + SEM. **d** Representative images of longitudinal cross-sections through ovaries of WT, *Puma*^−/−^ and *TAp63*^−/−^ female mice that had been treated with saline, cyclophosphamide or cisplatin. Scale bars = 200 µm **e**. Ovarian volume (µm^3^/ovary) of mice of the indicated genotypes 5 days following treatment (*n* = 4–5). Data are expressed as mean + SEM *p ≤ 0.05 compared with WT control (one-way ANOVA, Tukey’s multiple comparisons test)

Having established that the ovarian reserve is rescued by loss of PUMA or TAp63 following cyclophosphamide or cisplatin treatment, it was essential to assess whether the rescued oocytes were healthy and appeared normal. Morphological assessment of surviving primordial follicles across all treatments and genotypes showed no difference between those seen in saline-treated mice, and those which survived cisplatin or cyclophosphamide all appeared normal (Fig. 2a). However, in all mice in which significant primordial follicle depletion was observed (cyclophosphamide-treated WT and *TAp63*^−/−^ mice, and cisplatin-treated WT mice), small residual follicular structures containing granulosa cells were seen in the ovarian cortex (Fig. 2a). These structures have previously been identified as the remnants of primordial follicles^26,34^ and this suggests that cisplatin and cyclophosphamide directly cause primordial follicle depletion by initiating oocyte apoptosis. In contrast, granulosa cells appeared healthy, as evidenced by morphology and immunostaining for the granulosa cell marker FOXL2 (Fig. 2a; Fig. S4, Additional File 1).

### Atresia of growing follicles is not increased at day 5 post-treatment with cyclophosphamide or cisplatin

In addition to determining the numbers of healthy follicles, atretic follicle numbers were also evaluated. As described above, all primordial follicles remaining 5 days after treatment with the chemotherapeutic drugs appeared healthy and so this analysis was restricted to growing follicles (primary, secondary and antral). We found no significant differences in atretic follicle counts across genotypes and treatment groups (Figs. 2b, c). Assessment of ovarian volume (Figs. 2d, e) showed a statistically significant decrease in the ovaries of WT mice treated with cisplatin, and a nonsignificant trend toward reduction in WT mice treated with cyclophosphamide (WT saline: 3.15 × 10^9^ ± 0.42 × 10^9^ μm^3^ vs WT cisplatin: 1.54 × 10^9^± 0.50 × 10^9^ μm^3^, *p* < 0.05; vs WT cyclophosphamide 2.63 × 10^9^ ± 0.25 × 10^9^ μm^3^, *p* = 0.19). A reduction in ovarian volume is consistent with depletion of the ovarian reserve as evidenced by total follicle counts shown earlier (Fig. 1a).

### Oocytes rescued from chemotherapy by loss of PUMA or TAp63 are able to sustain normal fertility

After establishing that primordial follicles in *Puma*^−/−^ and *Tap63*^−/−^ mice surviving cyclophosphamide or cisplatin appeared morphologically normal, we then assessed whether they were capable of further development in order to support fertility and give rise to healthy offspring. To address this issue, we conducted a study in which WT, *Puma*^−/−^ and *TAp63*^−/−^ females were treated with saline, cyclophosphamide or cisplatin and continuously mated with WT males until females were deemed infertile or had reached 14 months of age.

**Fig. 3:**
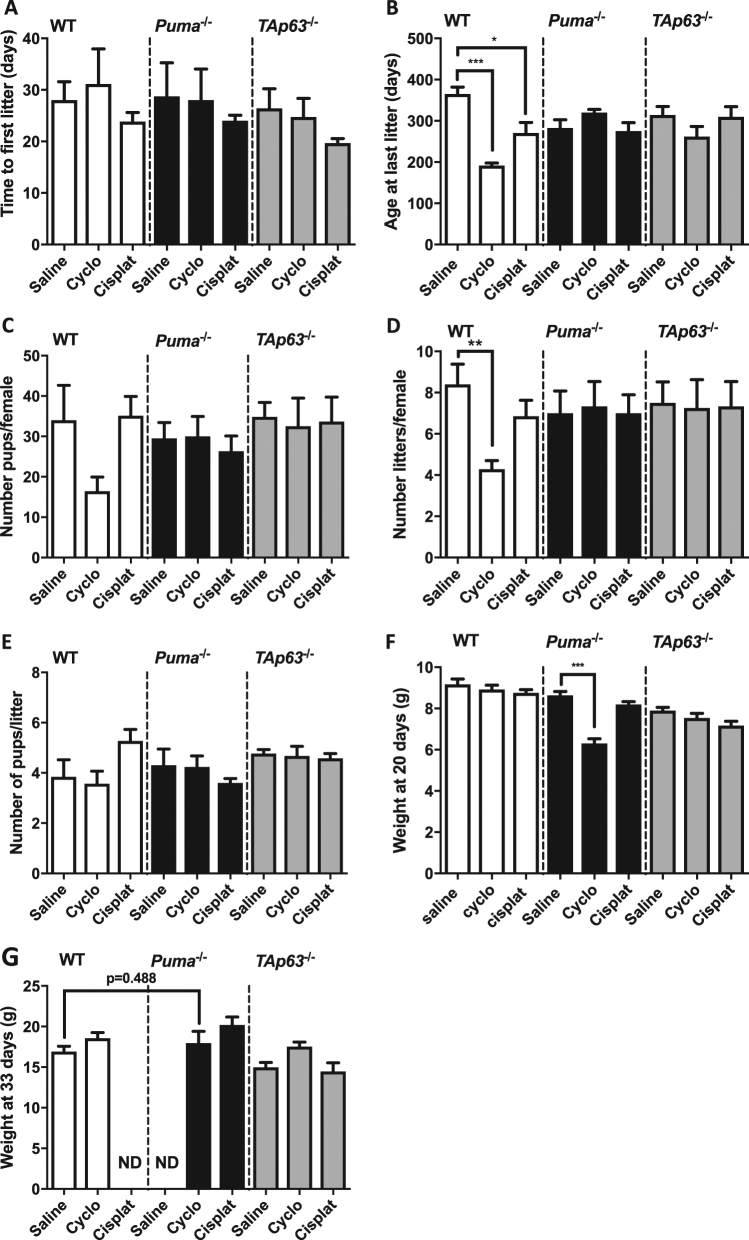
Fertility studies. WT, *Puma*^−/−^ and *TAp63*^−/−^ mice were treated with saline, cyclophosphamide or cisplatin and then mated continuously with untreated WT males of proven fertility. Time to first litter (**a**), age at last litter (**b**), total number of pups per female (**c**), total number of litters per female (**d**) and number of pups per litter (**e**) were assessed. Mean weight at weaning (**f**) and at 33 days (**g**) were evaluated in offspring as an overall indicator of gross health. ND not determined. Data are expressed as mean + SEM; *p ≤ 0.05, **p ≤ 0.01, ***p ≤ 0.001 (one-way ANOVA, Tukey’s multiple comparisons test).

In order to assess the total duration of fertility, two outcomes were measured: time to first litter (from first mating), and age at last litter. There were no differences between treatment groups in time to first litter (Fig. 3a), therefore age at last litter (Fig. 3b) can be regarded as an accurate marker of fertile lifespan. As expected given the massive depletion of primordial follicles, WT females treated with cyclophosphamide had a markedly shortened fertile lifespan as indicated by age at last litter (age at last litter: WT saline 365.2 ± 16.6 days vs WT cyclophosphamide 191.6 ± 6.1 days; *p* ≤ 0.0001). A smaller but still significant shortening of the fertile span was also observed in cisplatin-treated WT females (age at last litter: WT cisplatin 270.4 ± 25.8 days, *p* < 0.05). Correlating with this, there was a trend toward cyclophosphamide-treated WT females birthing fewer pups in total (WT saline 34 ± 8.6 pups vs WT cyclophosphamide 16 ± 3.5 pups; *p* = 0.11) (Fig. 3c) over fewer litters (WT saline 8.4 ± 0.98 litters vs WT cyclophosphamide 4.3 ± 0.42 litters, *p* = 0.0015) (Fig. 3d). Average litter size was not changed by treatment with cyclophosphamide or cisplatin compared with saline (WT saline 3.8 ± 0.7 pups/litter vs WT cyclophosphamide 3.6 ± 0.5 pups/litter; *p* = 0.75) (Fig. 3e).

Although fertility was clearly reduced in cyclophosphamide-treated WT females, the fertility of this group is remarkable considering that the total remaining follicle pool was only approximately 720 follicles per animal (i.e., a 90% reduction compared with saline-treated WT females) after treatment. It is also noteworthy that there was no difference in any of the outcomes measured in the fertility study between WT control females and WT females treated with cisplatin, although follicle numbers were reduced by 73% in the cisplatin-treated group. Collectively, these results show that there is remarkable conservation of fertile potential under conditions where the ovarian reserve has been significantly depleted, and even when almost completely destroyed.

### Offspring of cyclophosphamide- and cisplatin-treated females appear grossly healthy

Once it was clear that elimination of PUMA or TAp63 preserved fertility, it was essential to assess the health of offspring born to chemotherapy-treated mothers. Offspring of all genotypes, and across all treatments, appeared grossly normal at autopsy. Additionally, body weight at weaning was used as a measure of overall health (Fig. 3f; Fig S3, Additional File 1). Of note, pups of cyclophosphamide-treated *Puma*^−/−^ mothers were significantly lighter at weaning when compared with offspring of saline-treated *Puma*^−/−^ mothers. There was no difference between pups of female vs male sex (fig. S3). This effect was not seen in pups born to WT or *TAp63*^−/−^ females that had been treated with cyclophosphamide or cisplatin, nor was it seen in pups born to *Puma*^−/−^ mothers that had been exposed to cisplatin (Fig. 3f). Furthermore, when a cohort of these underweight pups were weighed again at 33 days, this difference had disappeared (Fig. 3g). This indicates that the initial weight disparity of the pups may have been due to maternal or nutritional factors.

## Discussion

In this study, we have made several novel findings: (1) PUMA is an essential apoptotic trigger for primordial follicle depletion following DNA damage induced by treatment with cyclophosphamide or cisplatin; (2) loss of PUMA completely protects fertility, (3) cyclophosphamide can deplete primordial follicle numbers independently of TAp63 although it is critical for the loss of primordial follicles after exposure to cisplatin; (4) there is significant inbuilt plasticity in the ovarian reserve, allowing for remarkable conservation of fertility, even in the event of massive primordial follicle loss.

We have previously identified PUMA as a crucial effector for TAp63-induced primordial follicle apoptosis in response to DNA damage caused by γ-irradiation^26^. In that study, elimination of PUMA alone was able to rescue 12–16% of the ovarian reserve from low and moderate doses of γ-irradiation, and higher levels of rescue were only achieved when PUMA plus a second pro-apoptotic BH3-only protein, called NOXA, were both lost^26^. In contrast, our current work shows that knockout of PUMA alone is sufficient to protect the entire primordial follicle pool against treatment with cyclophosphamide or cisplatin. This indicates that PUMA is a crucial effector of primordial follicle apoptosis in response to these DNA-damaging chemotherapeutic agents.

The specific underlying mechanisms by which different chemotherapeutic drugs exert their destructive effects in the ovary are still not known although several putative mechanisms have been suggested in previous animal studies. Direct toxicity may be caused by alkylators, which can cross the blood–follicle barrier and cause direct damage to the ovarian oocyte pool^35^. Indeed, multiple studies have found that cisplatin and cyclophosphamide both induce damage and apoptosis in primordial follicle oocytes^14,16,34,36^. However, some recent studies propose that cisplatin- and cyclophosphamide-induced loss at the primordial stage is not due to primordial follicle oocyte apoptosis, but another mechanism^37–40^. In those studies, primordial follicle loss in response to chemotherapy was attributed to the death of growing follicles, leading to the accelerated activation and maturation of primordial follicles via a phosphoinositide 3-kinase/protein kinase B/forkhead box protein O3a (PI3K/AKT/FOXO3a) pathway-dependent process, ultimately leading to a “burn-out” phenomenon^39,40^.

Although “burn-out” is one possible mechanism of follicle depletion, the results of our current study point to direct oocyte damage leading to PUMA-dependent apoptotic cell death being the predominant process involved in primordial follicle loss after treatment with cisplatin or cyclophosphamide. This conclusion is based on our finding that the dramatic loss of primordial follicles caused by either drug was not accompanied by a concomitant rise in the numbers of primary or growing follicles to suggest an activation effect, and overall, there was a massive net loss of total follicle numbers. Furthermore, in WT and *TAp63*^−/−^ mice treated with cyclophosphamide, and in WT mice treated with cisplatin, we histologically identified the frequent occurrence of cortical primordial follicle remnants, where a follicular structure consisting of surviving granulosa cells remained, but without a central oocyte. This is similar to what was observed in our previous study in which such remnants were seen following γ-irradiation-induced oocyte damage^26^. This is also consistent with a previous detailed study examining the ovotoxicity of cyclophosphamide in mice, which also identified a massive net loss of follicles without a rise in growing follicles^34^. Overall, these observations argue strongly in favor of direct oocyte damage and apoptosis being the primary process for oocyte depletion after chemotherapy.

Marked differences were seen between the protection conferred by TAp63 loss versus PUMA knockout in response to treatment with cyclophosphamide, with only the latter being fully protective. This suggests that the TAp63-induced pathway of oocyte apoptosis after DNA damage is not the predominant one activated following the genotoxic insult caused by cyclophosphamide. Other transcriptional activators for *Puma* have been identified. For example, p53, widely recognized as the “guardian of the genome”^41^, has been investigated regarding a possible role in primordial follicle oocyte apoptosis. Livera and colleagues found that p53 was not expressed in the nuclei of small oocytes^42^. Furthermore, we found that TAp63, but not p53, was essential for DNA damage-induced transcriptional induction of PUMA in primordial follicle oocytes following γ-irradiation^26^. To the contrary, however, a subsequent study showed that p53 was highly expressed in the nuclei of primordial and primary follicle oocytes after 48 h of cisplatin treatment in vitro^16^. Thus, in this context p53 may have a role to play in the transcriptional induction of PUMA in response to cyclophosphamide when TAp63 is absent. This may indicate that treatment with cyclophosphamide can induce p53 expression.

**Fig. 4:**
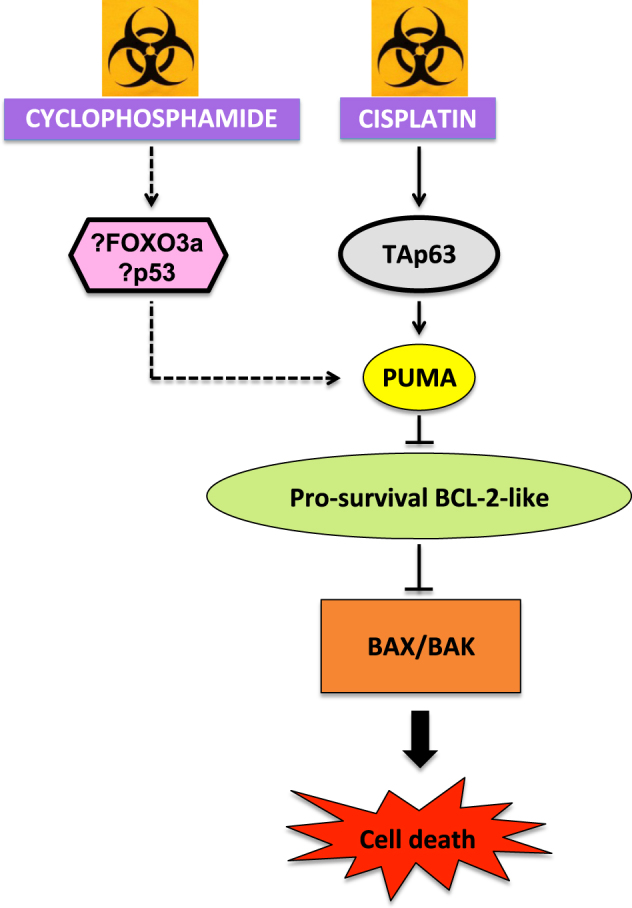
Pathways to oocyte apoptosis following DNA damage induced by cyclophosphamide and cisplatin. DNA damage caused by cisplatin activates a signaling pathway in which TAp63 plays a key regulatory role, with downstream transcriptional induction of *Puma*, resulting ultimately in the unleashing of the apoptosis effectors BAX and BAK. Cyclophosphamide-induced DNA damage activates an alternate signaling pathway that does not require TAp63; possible mediators are FOXO3a and/or p53. Solid arrows denote known pathways. Dashed arrows denote possible alternative pathways (not yet characterized)

Another potential transcriptional activator of PUMA in the ovary is FOXO3a. A study examining somatic cell lines showed that FOXO3a is able to directly bind to a site in the *Puma* promoter and activate its expression in a p53-independent manner^43^. FOXO3a is expressed in primordial follicle oocytes and is important for the suppression of follicular activation^44–46^. Additionally, phosphorylation (and thus functional suppression) of FOXO3a is associated with inhibition of oocyte apoptosis^47^. Other studies have shown that cyclophosphamide and cisplatin can both activate the PI3K/phosphatase and tensin homolog (PTEN)/AKT signaling pathway, resulting in increased FOXO3a phosphorylation in oocytes^37,39^. Thus, FOXO3a may have a dual role in primordial oocyte depletion caused by cyclophosphamide, exerting a pro-activation effect on primordial follicles as part of the “burn out” process, whereas also forming part of a candidate pathway that triggers apoptosis in oocytes through transcriptional induction of *Puma* (Fig. 4).

Long-term fertility trials conducted during this study showed that the rescued pool of primordial follicles in PUMA-deficient mice was able to give rise to normal sustained fertility. Assessment of offspring health showed that pup health was not compromised by either the treatment given, or genotype. Although there was an initial difference in weight at weaning between offspring of saline- vs cyclophosphamide-treated *Puma*^−/−^ mothers, this effect had disappeared by 33 days, suggesting maternal/nutritional factors rather than problems intrinsic to PUMA deficiency. Overall, our data suggest that oocytes sustaining DNA damage due to treatment with these two chemotherapeutic agents are capable of effective DNA repair in the absence of the powerful pro-apoptotic action of PUMA. However, further assessment of offspring health is warranted, given that at least one study in mice reports the transgenerational transmission of developmental side-effects in offspring after maternal exposure to chemotherapy^48^. Similarly, several studies have demonstrated that exposure of male mice to commonly used chemotherapeutic agents (cyclophosphamide, mitomycin C and procarbazine) and ionizing radiation induces germline mutations, thereby increasing the frequency of mutations and defects in their offspring^49,50^. These data raise important issues concerning delayed transgenerational effects in the children of survivors of anticancer therapy, which require comprehensive assessment if inhibition of apoptosis is to be seriously considered as a therapeutic approach.

Our findings that treatment with cyclophosphamide and cisplatin shortened the fertile lifespan in WT female mice were unsurprising, given what is already known about the deleterious effects of these drugs on human female fertility and their relative gonadotoxicities. In keeping with the complete preservation of primordial follicle numbers seen in *Puma*^−/−^ females treated with either agent, we saw a corresponding preservation of fertility. This suggests that rescued oocytes are indeed able to sustain fertility across the entirety of the normal fertile lifespan in these mice. However, the implications of our fertility trials reach beyond the preservation of fertility by the inhibition of apoptosis. Remarkably, WT mice treated with cyclophosphamide were still able to produce offspring over a number of litters, despite having received a dose of cyclophosphamide that destroyed 96% of their primordial follicles. This striking finding indicates that when severe follicle depletion has occurred, the dynamics of the primordial follicle pool alter to maximize efficiency and minimize waste, in order to optimize fertile potential. This is possibly achieved by restricting the rate of primordial follicle activation and/or reduced loss of growing follicles, which normally occur at relatively high levels. This is in keeping with retrospective cohort studies of women who have undergone unilateral oophorectomy showing that the age at menopause was brought forward by only 1.1–1.2 years despite significant loss of the ovarian reserve^51,52^.

An effective inhibitor of PUMA has not yet been developed. However, checkpoint kinase 2 (CHK2) and the executioner kinase, CK1, have recently been identified as critical players in the elimination of mouse oocytes following double-stranded breaks in DNA^53,54^, with their function being essential for the downstream activation of *TAp63*^55^. CHK2-deficient mice are resistant to γ-irradiation-induced oocyte loss, even in the presence of TAp63^53^, and inhibition of CHK2 and CK1 is effective in vitro in rescuing oocytes from TAp63-mediated apoptosis induced by γ-irradiation, cisplatin and the anthracycline, doxorubicin^56,57^. Thus, targeting kinases acting upstream of *TAp63* offers promise in developing fertoprotective strategies against at least in context of these two chemotherapeutic drugs and γ-irradiation.

In summary, this study demonstrates that PUMA is a key initiator of apoptosis in primordial follicle oocytes in mice following treatment with a single dose of cyclophosphamide or cisplatin. Loss of PUMA alone rescues 100% of the ovarian reserve following treatment with either drug, although the pathways by which *Puma* is transcriptionally activated may differ between them, with cisplatin activating a TAp63-dependent process but cyclophosphamide acting via a TAp63-independent pathway. Crucially, we have shown that this translates to a complete preservation of fertile potential and the fertile lifespan in the *Puma*^−/−^ females, with no obvious ill effects on offspring. Collectively, these data further strengthen the argument that inhibition of oocyte apoptosis may be a promising potential approach of fertility preservation in females following DNA-damaging cancer therapies.

## Materials and methods

### Mice

The generation and genotyping of *Puma*^*−/−*^^58^ and *TAp63*^−/−^^59^ mice on a C57BL/6 background have been previously described. Mice were kept in a photo-controlled animal facility (12-h light–dark cycle) with free access to commercial feed and tap water.

### Injection of mice

For follicle enumeration, postnatal day 50 mice received a single intraperitoneal injection of saline, cisplatin (5 mg/kg), or cyclophosphamide (300 mg/kg) (*n* = 5/treatment/genotype). Mice were culled 5 days later, and ovaries harvested and fixed in Bouins solution. For fertility trials, female mice were treated as above (*n* = 7–9/treatment/genotype), then kept for breeding.

### Follicle quantification

Bouin’s-fixed ovaries were embedded in glycomethacrylate, cut into 20 μm sections, stained with periodic acid–Schiff, and counterstained with hematoxylin. Stereological quantification of primordial and primary follicles was performed using the 100 × oil immersion objective on an Olympus BX50 microscope (Tokyo, Japan) equipped with an Autoscan stage (Autoscan Systems Pty Ltd, Melbourne, Victoria, Australia) in conjunction with the Stereo Investigator stereological system (Version 11.06.02, MBF Bioscience 2015, MicroBrightField, Inc., Williston, Vermont, USA), by evaluating every 6th section using stereological methods previously described in detail^60^. Secondary, antral and atretic follicles were counted every 9th section, then multiplied by a factor of 9 to obtain an estimated total count per ovary, then by 2 to obtain an estimated total count per animal. Corpora lutea were quantified by direct counting of every 3rd section to avoid duplicate counts. Follicles were classified as described in Fig. S1, Additional File 1.

### Ovarian volume

Ovarian volume was estimated stereologically using the Cavalieri Estimator function on the Stereo Investigator software. A point-counting grid was used to estimate the area of every 3rd section through the ovary, with the density of this grid (200 μm) calculated to obtain an appropriate coefficient of error (CE) according to the formula proposed by Gundersen and Jensen^61^.

### FOXL2 immunohistochemistry

Ovaries were fixed with 10% neutral-buffered formalin for 24 h, processed in ethanol, embedded in paraffin and serially sectioned at 5 μm intervals. Nine sections (three sections per slide; three slides per ovary, taken from the middle and edges of the ovary) were analyzed from each of three ovaries. Sections were de-paraffinized, rehydrated and subjected to microwave antigen retrieval in sodium citrate buffer (pH 6.0) for 10 min. Sections were then blocked with 5% goat serum for 1 h at room temperature, then incubated with primary antibody at 4 °C overnight. The primary antibody used was rabbit anti-FOXL2 (courtesy of Dr Dagmar Wilhelm, The University of Melbourne) used at 1:300. After washing with tris-NaCl-Tween buffer (TNT), slides were incubated with biotinylated goat secondary antibodies against rabbit IgG for 30 min at room temperature, then following a thorough TNT wash, with Vectastain for 30 min. Slides were then washed with TNT again prior to being incubated with 3, 3'-diaminobenzidine, then counterstained with Harris hematoxylin, washed with acid alcohol, then “blued” using lithium carbonate. Slides were then dehydrated, mounted with dibutylphthalate polystyrene xylene, and visualized using light microscopy.

### Fertility study

Drug- or vehicle-treated female mice (*n* = 7–9/treatment/genotype) were mated with proven C57BL/6 WT males, and kept for breeding until they reached 14 months of age, or until no litters had been produced for ≥ 3 months, whichever was earlier. For analysis, time to first litter, age at last litter, total number of litters per female, total number of pups per female, litter size and gross observations of pup morphology were recorded. Animals were eliminated from analyses of fertile lifespan if they were culled or died prior to completion (see Table S1, Additional File 1, for a summary of animals used in this study). Pups were weighed at weaning (PN21) and a smaller cohort were also weighed at PN33.

### Statistical analysis

Data are shown as mean ± SEM and statistical analysis was undertaken using GraphPad Prism (GraphPad Software, Inc., La Jolla, CA, USA). Data were analyzed using Student's *t*-test for pairwise comparisons, or where multiple comparisons were required, one-way analysis of variance (ANOVA) with significance determined by Tukey’s post-hoc test. Differences were considered statistically significant when *p* ≤ 0.05.

## Electronic supplementary material

Supplemental Figures 1-4, Table S1
