# Supplementary material for: Loss of PUMA protects the ovarian reserve during DNA-damaging chemotherapy and preserves fertility
Source: Cell Death Dis. 2018 May 23;9(6):618. doi: 10.1038/s41419-018-0633-7 (PMC5966424; doi:10.1038/s41419-018-0633-7)
Supplement: Supplementary file 1 — Supplemental Figures 1-4, Table S1 [file 41419_2018_633_MOESM1_ESM.docx]

**Figure S1. Assessment of follicular stage**

**
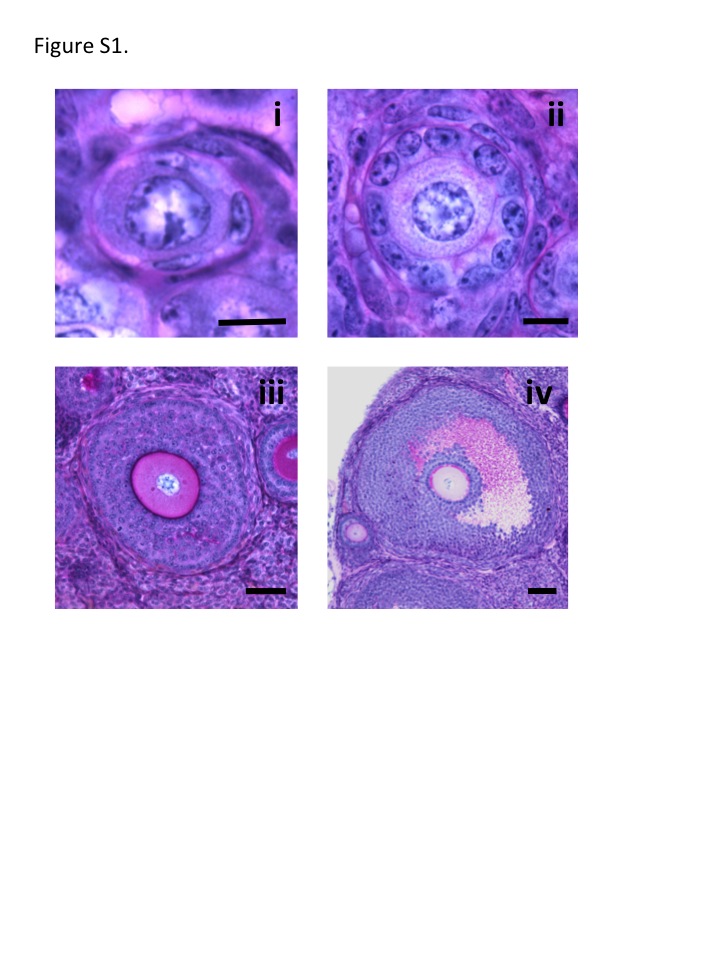
**

Representative images of PAS-stained sections showing follicles in the primordial **(i)**, primary **(ii)**, secondary **(iii)**, and antral **(iv)** stages. Scale bar = 10 μm in (i) and (ii); scale bar = 50 μm in (iii) and (iv).

**Figure S2. Corpus luteum quantification**

**
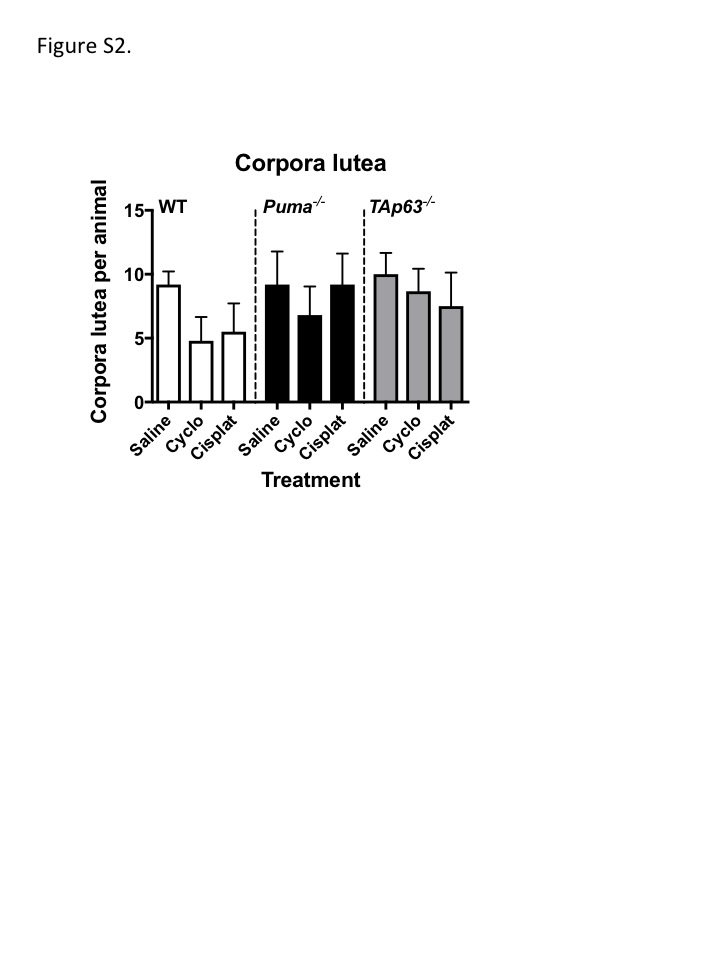
**Corpus luteum quantification per animal of the indicated genotypes after treatment with saline (control), cyclophosphamide or cisplatin. Data are expressed as mean+SEM.

**Figure S3. Offspring of *Puma*^-/-^ females: male *vs* female pup weights at weaning**

**
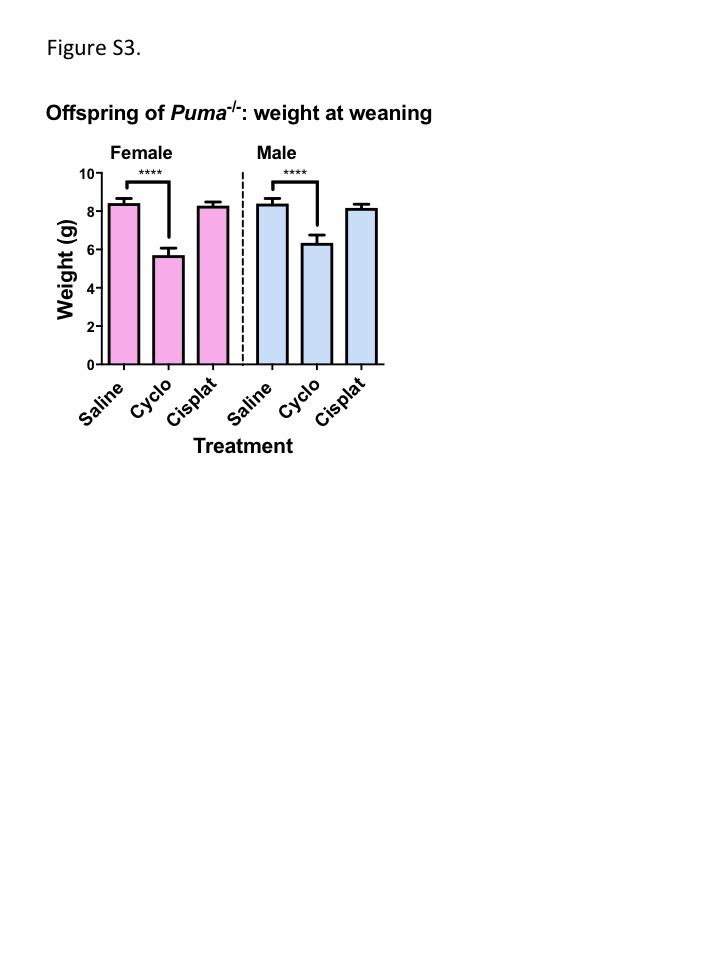
**

Weight at weaning of offspring of *Puma*^-/-^ females that had been treated with saline, cyclophosphamide or cisplatin. Data are expressed as mean+SEM ****P<0.0001 (one-way ANOVA, Tukey’s multiple comparisons test).

**Figure S4. FOXL2 immunohistochemistry on follicular remnants in ovaries of cyclophosphamide-treated WT females**

**
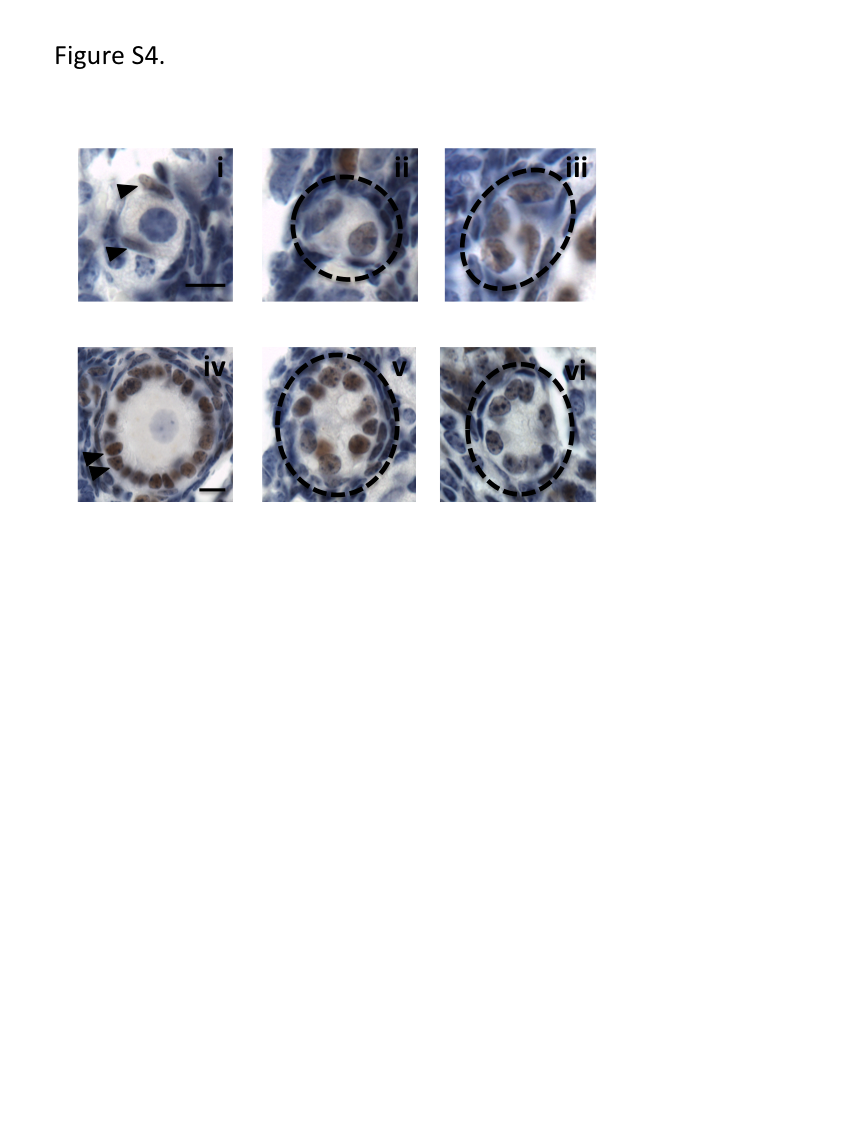
**

Representative images of FOXL2 immunohistochemistry in ovaries of WT mice that had been treated with cyclophosphamide, showing granulosa cells in a surviving primordial follicle **(i)**, primordial follicle remnants **(ii, iii)**, a surviving primary follicle **(iv)**, and primary follicle remnants **(v, vi)**. Arrowheads denote individual granulosa cells; dashed circles indicate follicular remnants. Scale bar = 10 μm.

**Table S1. Animals used in fertility study**

| Genotype | Treatment | Total # | # Fertile* (%) | # Assessed For Fertility Outcomes** |
| --- | --- | --- | --- | --- |
| WT | Saline | 7 | 7/7 (100) | 5/7 |
|  | Cisplatin | 8 | 8/8 (100) | 6/8 |
|  | Cyclophosphamide | 9 | 9/9 (100) | 7/9 |
|  |  |  |  |  |
| *Puma^-/-^* | Saline | 6 | 5/6 (83) | 4/5 |
|  | Cisplatin | 7 | 6/7 (86) | 3/6 |
|  | Cyclophosphamide | 7 | 6/7 (86) | 6/6 |
|  |  |  |  |  |
| *TAp63^-/-^* | Saline | 7 | 7/7 (100) | 5/7 |
|  | Cisplatin | 6 | 4/6 (67) | 3/4 |
|  | Cyclophosphamide | 7 | 7/7 (100) | 4/7 |
|  |  |  |  |  |

* Produced at least 1 litter

*Indicates the number of animals used (of those fertile) to assess total pup numbers, litter number and fertile lifespan. Mice were eliminated from this analysis if they died prior to the conclusion of the study. Reasons for death included unknown causes (normal at autopsy), killed by male, birthing difficulties (dystocia), mastitis and prolapse. Causes of death were distributed amongst all genotypes and treatments, with no particular association with any one genotype or treatment.
